# Pulmonary medium vessel vasculitis in an 11 year old boy: Hughes Stovin syndrome as a variant of polyarteritis nodosa?

**DOI:** 10.1186/1546-0096-9-19

**Published:** 2011-08-04

**Authors:** Willemien de Vries, Gerard H Koppelman, Marc TR Roofthooft, Hendrika Bootsma, Martha K Leijsma, Wineke Armbrust

**Affiliations:** 1 Department of Pediatrics, Beatrix Children's Hospital, University Medical Centre, University of Groningen, The Netherlands; 2 Department of Pediatric Pulmonology and Pediatric Allergology, Beatrix Children's Hospital, University Medical Centre, University of Groningen, The Netherlands; 3 Department of Pediatric Cardiology, Beatrix Children's Hospital, University Medical Centre, University of Groningen, The Netherlands; 4 Department of National Expertise Centre for Children with Pulmonary Hypertension, Beatrix Children's Hospital, University Medical Centre, University of Groningen, The Netherlands; 5 Department of Pediatric Rheumatology, Immunology and Infectious Disease, Beatrix Children's Hospital, University Medical Centre, University of Groningen, The Netherlands; 6 Rheumatology and Clinical Immunology, University Medical Centre Groningen, University of Groningen, The Netherlands

## Abstract

We present the case of an 11-year-old boy presenting with haemoptysis, dyspnoea and weight loss as a manifestation of isolated pulmonary vasculitis, leading to pulmonary hypertension. He also appeared to have a longstanding dural venous sinus thrombosis. This rare presentation, especially in childhood, might represent a case of the seldomly reported Hughes-Stovin syndrome. The patient achieved remission after therapy with cyclophosphamide pulses and high-dose steroids. Based on the presented case and review of the literature, we propose that this syndrome might be a variant of polyarteritis nodosa. This report highlights diagnostic issues and describes a successful treatment regimen.

## Background

According to the EULAR/PRINTO/PReS consensus criteria vasculitis of childhood is classified as small, medium-sized or large vessel vasculitis, or 'other' [1]. In childhood, pulmonary involvement is most frequently observed in vasculitis which preferentially affects small vessels, such as Wegener's granulomatosis, microscopic polyangiitis and Churg-Strauss syndrome. Pulmonary involvement is seldomly observed in large and medium vessel vasculitis, such as Takayasu arteritis, Kawasaki disease and polyarteritis nodosa (PAN) [1-3]. An exception is Behçet's disease (BD), in which the formation of pulmonary artery aneurysms occurs in 1-8% of adult BD patients. Similar rates have been found in pediatric case series [2,4-6].

In this report we describe an eleven-year-old boy suffering from pulmonary medium sized vessel vasculitis, combined with dural venous sinus thrombosis. We highlight the difficulty in establishing a definite diagnosis in the context of current diagnostic criteria and discuss Hughes-Stovin syndrome (HSS) as a potential diagnosis. HSS is characterized by the combination of multiple bilateral pulmonary artery aneurysms and venous thrombosis, and has been suggested to be an incomplete variant of BD [5,7]. We will review pediatric cases of HSS from the literature and discuss the possibility that it is rather a variant of PAN with specific pulmonary involvement, based on its clinical manifestations in relation to current diagnostic guidelines and its therapeutic response.

## Case Presentation

An 11-year-old boy was admitted to our hospital with cough, fatigue, and weight loss (~4 kg) of 4 months duration, and hemoptysis starting the day before admission. The past medical history was unremarkable. His mother was Spanish, his father Dutch. The family medical history was negative for autoinflammatory or autoimmune diseases.

Clinical examination was unremarkable other than a pale appearance. No oral or genital ulcers were present. Laboratory examination revealed elevated inflammatory parameters (ESR 59 mm/h (normal, 3-13 mm/h); CRP 69 mg/L (normal < 5 mg/L); leucocytes 12.2*10^9^/L (normal, 4.0-10.0*10^9^/L); microcytic anemia (Hemoglobin 6.2 mmol/L (normal, 6.6-9.3 mmol/L); MCV 65.9 fl (normal, 80-94 fl)); and normal renal function (serum creatinin 42 μmol/L (normal, 31-68 μmol/L), urinary protein excretion 0.1 g/24 h (normal, < 0.3 g/24 h)). Tuberculin skin test, pathergy test, plasma lupus anticoagulant, anti-cardiolipins, direct Coombs test, angiotensin converting enzyme, ANA and ANCA were negative.

A chest X-ray showed a broad mediastinum, without pulmonary abnormalities. A CT-scan showed an enlarged subcarinal mediastinal mass branching towards both pulmonary hili, without parenchymal disease. The patient developed febrile episodes every afternoon (up to 39°C), and complained of mild myalgia. Diagnostic considerations included atypical pneumonia, tuberculosis, lymphoma, sarcoidosis, autoimmune and autoinflammatory disorders.

A bronchoscopy with transbronchial biopsy and bronchial alveolar lavage was performed. The biopsy results were not consistent with malignancy or sarcoidosis but were not conclusive. Therefore, a cervical mediastinoscopy was performed, and histological examination of the enlarged lymph node revealed no signs of malignancy, tuberculosis or sarcoidosis, but a reactive pattern with numerous lymphocytes, lymphoblasts and plasma cells. Culture of sputum, blood, stomach contents, bronchoalveolar lavage fluid and serology could not identify an infectious cause explaining the reactive lymphadenopathy.

**Figure 1:**
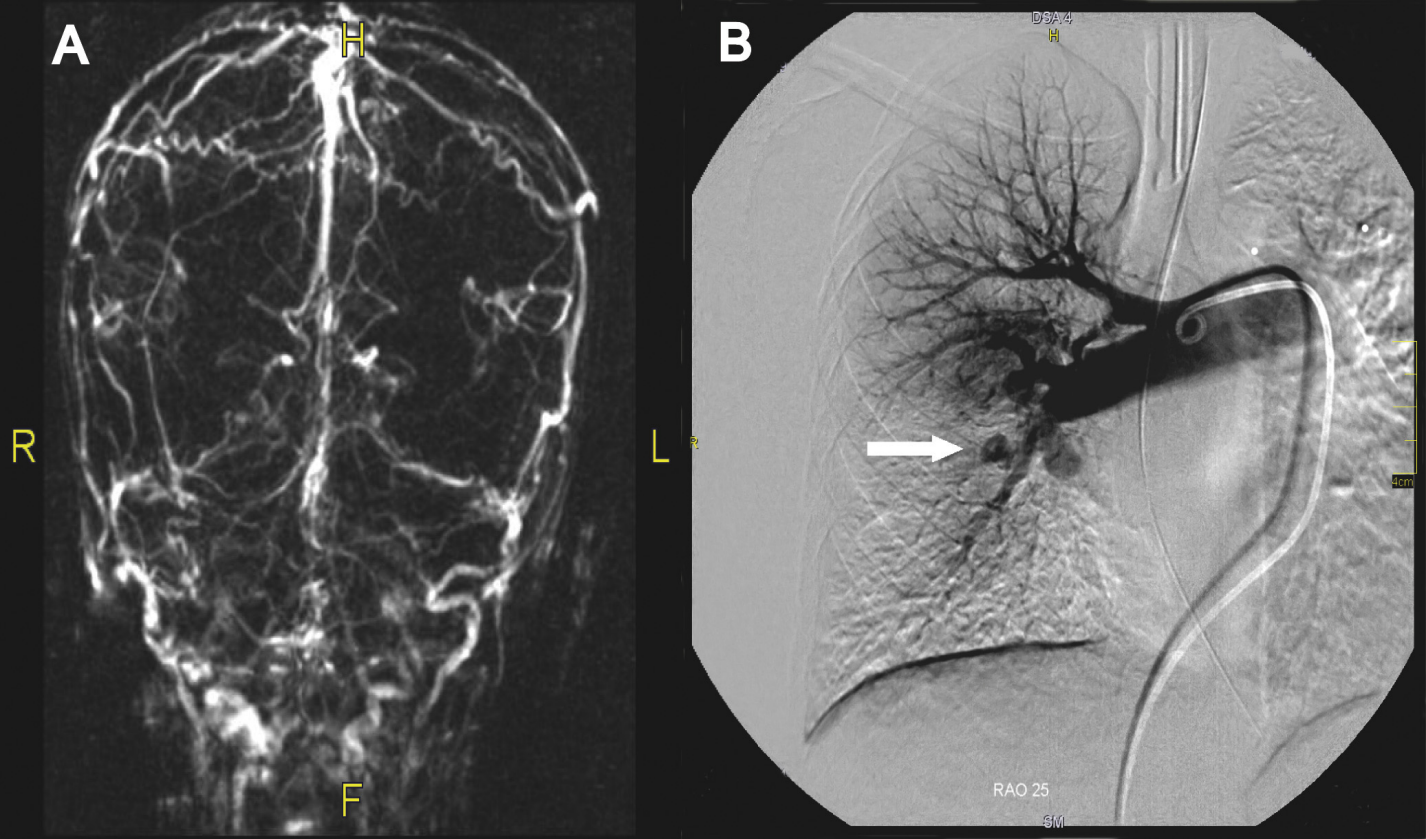
**Figure 1: Imaging studies demonstrating pulmonary aneurysms and venous sinus thrombosis. **A)Magnetic resonance venography of the venous sinus, demonstrating extensive formation of venous collaterals.B)Angiography of the right pulmonary artery. The angiography demonstrates multiple aneurysms (arrow), enlargement of central pulmonary arteriesand abruptly diminished calibre of peripheral pulmonary vessels.

Evaluation by an ophthalmologist for autoimmune related eye disease showed bilateral papilledema. There were no further symptoms of a raised intracranial pressure. Magnetic resonance imaging of the cerebrum showed dural venous sinus thrombosis with extensive collateral vessel formation (Figure 1A). Six weeks after admission, the patient again developed hemoptysis. Physical examination now revealed a cardiac murmur, due to tricuspid regurgitation, and a loud second heart sound, suggesting pulmonary hypertension. This was confirmed by cardiac catheterization. The mean pressure in the left pulmonary artery was 30 mmHg, the pulmonary wedge pressure was 13 mmHg, the cardiac index was 2 l/min/m^2^, and the pulmonary resistance measured 8.4 resistant units per m^2^, thus indicating moderate pulmonary hypertension. Angiography and CT-angiography showed multiple aneurysms in the medium sized arteries (pulmonary artery, Figure 1B). The abdominal, cervical and coronary arteries were also investigated by angiography and appeared unaffected. The performance of an open lung biopsy was considered too risky because of the patient's clinical condition.

Under the working diagnosis of medium-sized vessel vasculitis with moderate pulmonary hypertension as a consequence, the patient was treated with intravenous gamma globulin (2 g/kg) for two days, intravenous prednisolone (35 mg/day) and cyclophosphamide (750 mg/m^2^, once per month, 12 pulses), sildenafil (40 mg/day) and bosentan (125 mg/day). Anticoagulation therapy was initiated in the acute phase but stopped after one week. Ten days thereafter the patient developed recurrent hemoptysis.

Twenty-five days after initiation of the anti-inflammatory treatment, the ESR returned to the normal range; however, after five weeks the patient developed massive bleeding in his respiratory tract. The aneurysmal right bronchial artery was identified as the focus of the bleeding by digital substraction angiography and embolized with microspheres. The patient required mechanical ventilation and was treated in the intensive care unit for three weeks. After ten weeks he was sufficiently recovered to be discharged. The exercise tolerance was severely decreased but improved gradually as demonstrated by the results of the 6 minutes walk test. Seven weeks after embolization, the walked distance was 178 meters; eighteen months later this had improved to 442 meters. However, the walked distance is still below reported results from healthy age-matched males [8].

After the final cyclophosphamide pulse azathioprine was started (2 mg/kg/day, for 2 years). Steroids were tapered gradually and discontinued 18 months after initiation of therapy. No important side effects of the medication occurred. Currently, after eighteen months of treatment, the patient is still in remission. The inflammatory parameters are not elevated and fever is absent. Upon re-evaluation on CT scan, there were only minor residual vessel wall derangements visible at the site of the former aneurysms. The follow-up of the pulmonary hypertension was done indirectly by cardiac ultrasonography and showed no increase of pulmonary hypertension. Sildenafil and bosentan are continued. The papilledema had resolved, cerebral imaging was not repeated.

## Discussion

We present a case of recurrent hemoptysis and pulmonary hypertension in an 11-year-old boy. The symptoms of the patient appeared to be the result of an inflammatory destructive process solely affecting the medium sized pulmonary and bronchial vessels. However, the involvement of both pulmonary and systemic vessels makes classification of the disease difficult.

The vasculitis of medium-sized vessels described in this report might be classified as PAN because the patient met the full diagnostic criteria of childhood PAN, that include: angiographic or histologic abnormalities combined with myalgia, and symptoms suggesting involvement of a major organ system [1].

PAN is a vasculitis typically involving medium sized vessels and the disease is rare in childhood with an incidence of < 1 in 1 million [9]. PAN most commonly involves skin, joints, peripheral nerves, gastrointestinal tract and kidney, and disease manifestations are diverse. Pulmonary involvement is rare but was described to occur in ~11% of juvenile systemic PAN cases [10]. In the same study, 8 out of 56 (14%) juvenile PAN patients had aneurysms in central nervous system arteries. Pulmonary involvement in PAN includes arteritis of bronchial arteries, but diffuse alveolar damage and interstitial fibrosis have also been detected in autopsy cases [11]. The risk of venous thrombosis in PAN is higher compared to the general population [12,13], which may be attributable to the systemic inflammation. In the presented case, all prothrombotic factors examined (lupus anticoagulant, anti-cardiolipins) were negative. To our knowledge, the incidence of venous sinus thrombosis in PAN has not been studied.

HSS is characterized by multiple bilateral pulmonary artery aneurysms and venous thrombosis [5,7,14]. Hughes and Stovin described this syndrome for the first time in 1959. To our knowledge, only four pediatric cases have been described, aged 12, 14 (twice) and 16 (Table 1) [14,15]. The pathogenesis of this disease remains speculative, and previously it has been suggested that HSS is an incomplete variant of BD based on the occurrence of pulmonary vasculitis and venous thrombosis in both disease entities [5,7]. Review of adult and pediatric cases showed that no history of oral ulcers has been described in the context of HSS. That is mandatory for the diagnosis of BD as the diagnostic criteria include oral ulcers combined with two of the following symptoms: genital ulcers, ocular lesions, skin lesions, positive pathergy test (Table 1) [4,6,16].

**Table 1:** Pediatric cases of Hughes Stovin Syndrome

Author	Gender	Age	Symptoms	Oral/Genital ulcera	Myalgia	Conjunctivitis	Histology/angiography	Therapeutic regimen
Hughes [14]	m	14	PAA, CVST	nr	nr	nr	+	
	m	14	PAA, CVST, DVT	nr	nr	+	+	prednisolone
Roberts [15]	m	12	PAA, DVT	nr	nr	nr	+	surgery
Emad [7]	m	16	PAA, CVST, DVT	-	nr	-	+	prednisolone, azathioprin
This report	m	11	PAA, CVST	-	+	-	+	cyclophosphamide prednisolone

m: male; PAA: pulmonary artery aneurysms; CVST: cerebral venous sinus thrombosis; DVT: deep venous thrombosis;

nr: not reported; + symptom present; - symptom absent.

We argue, given the symptoms described in the current and previous case reports, that it is more likely that HSS is a variant of PAN rather than BD. This would apply to children and adults, as the diagnostic criteria do not differ in this respect. The presented case fulfills the diagnostic criteria with angiographic abnormalities of medium-sized vessels, and myalgia. In other published HSS cases, the presence or absence of mylagia or one of the other minor criteria for the diagnosis of PAN, are not mentioned. The therapeutic approach to HSS is similar to that of PAN, as both respond well to cyclophosphamide courses [17]. Our case illustrates the beneficial effect of a treatment regimen entailing a combination of high-dose steroids and cyclophosphamide pulses.

## Conclusions

In summary, we present a case of isolated pulmonary vasculitis with involvement of both the pulmonary and bronchial vessels and concomitant dural venous sinus thrombosis in an 11-year-old boy. The presentation of pulmonary vasculitis with lymphadenopathy, fever, weight loss, and hemoptysis as presented in this report has a broad differential diagnosis, including infections, malignancies and inflammatory disease. This may well represent a case of HSS. Based on our findings and previous case reports we argue that HSS might be a disease closely related to PAN. We cannot support this speculation with histological findings, since it was impossible to obtain histology in the presented patient. Treatment with high-dose steroids and cyclophosphamide induced remission and thus might be an effective therapeutic regimen in (pediatric) cases of HSS.

## Consent

Written informed consent was obtained from the patient's parents for publication of this case report and any accompanying images. A copy of the written consent is available for review by the Editor-in-Chief of this journal

## List of abbreviations

PAN: polyarteritis nodosa; BD: Behçet's disease; HSS: Hughes-Stovin syndrome; ESR: Erythrocyte sedimentation rate; CRP: C-reactive protein; MCV: Mean corpuscular volume; ANA: Anti-nuclear antibodies; ANCA: Anti-neutrophil cytoplasmic antibodies; CT: computed tomography; PAA: pulmonary artery aneurysms; CVST: cerebral venous sinus thrombosis; DVT: deep venous thrombosis

## Competing interests

The authors declare that they have no competing interests.

## Authors' contributions

WdV, GK, MR, HB, ML, WA contributed to the diagnostic work-up and treatment of the patient. WdV, GK, MR and WA have been involved in drafting the manuscript. All authors read and approved the final manuscript.
